# Can we achieve universal health coverage without a focus on disability? Results from a national case-control study in Guatemala

**DOI:** 10.1371/journal.pone.0209774

**Published:** 2018-12-27

**Authors:** Hannah Kuper, Islay Mactaggart, Carlos Dionicio, Rafael Cañas, Jonathan Naber, Sarah Polack

**Affiliations:** 1 International Centre for Evidence in Disability, London School of Hygiene & Tropical Medicine, London, United Kingdom; 2 National Council on Disability, Guatemala City, Guatemala; Indiana University Purdue University at Indianapolis, UNITED STATES

## Abstract

**Objective:**

To compare access to healthcare services for people with disabilities to those without disabilities, within a national case-control study in Guatemala.

**Methods:**

We undertook a population-based case-control study, nested within a national survey in Guatemala. Cases with disabilities were people with self-reported difficulties in functioning. One control without disabilities was selected per case, matched by age, gender and cluster. Information was collected on: health status, access to health services and rehabilitation, and socioeconomic status.

**Results:**

The study included 707 people with disabilities, and 465 controls. People with disabilities were more likely to report a serious health problem (aOR 2.8, 2.2–3.7) or doctor-diagnosis of one of 17 general health conditions (aOR 2.9, 2.2–3.8) as compared to controls without disabilities. People with disabilities were twice as likely as controls to have received treatment for a diagnosed condition (aOR 2.2, 1.7–2.8). Coverage of treatment for impairment-related health conditions was low, as was awareness and access to rehabilitation services. People with disabilities were more likely than controls to report being disrespected (aOR 1.9, 1.0–3.7) or finding it difficult to understand information given (aOR 1.6, 1.1–1.4).

**Conclusion:**

Efforts are needed to raise awareness about rehabilitation services and improve quality of health services for people with disabilities in Guatemala, to ensure that their rights are fulfilled and to assist in the achievement of Universal Health Coverage. Better tools are needed to measure healthcare access, including consideration of geographic access, quality and affordability, to allow the generation of comparable data on access to healthcare among people with disabilities.

## Introduction

Achieving Universal Health Coverage (UHC) is an important target of the Sustainable Development Goals. [1] UHC means that people have access to all the services that they need, including the full range of promotive, preventive, curative and rehabilitative health services. [2] These services must be of sufficient quality to be effective, and be available to people without incurring financial hardship. There is increasing awareness that marginalised groups, including people with disabilities, must be fully included in efforts to achieve UHC,[1] as they make up a large proportion of the population and may have different or greater healthcare needs.

People with disabilities include those who have long-term physical, mental, intellectual or sensory impairments which in interaction with various barriers may hinder their full and effective participation in society on an equal basis with others.[3] Globally, the WHO estimates that there are 1 billion people with disabilities, constituting 15% of the world’s population.[4] By definition, people with disabilities have an impairment (e.g. diabetic-retinopathy related visual impairment), and impairments arise from health conditions (e.g. diabetes). Therefore, people with disabilities will have health care needs due to their impairment and underlying health condition. Additionally, people with disabilities may be more vulnerable to poor health through other pathways, including their increased risk of poverty and vulnerable living conditions, exclusion from health services, and higher prevalence of adverse health behaviours.[5] [6] Consequently, people with disabilities will on average have higher general healthcare needs overall,[4, 7–9] including for health promotion[6], general health care, and rehabilitation and specialist treatment related to their underlying impairment.[10] These latter services include medication, surgery, assistive devices, and therapeutic rehabilitation (e.g. physical therapy).

Access to healthcare is often viewed in a narrow sense, with a focus on geographic accessibility. A broader conceptualization of access is preferable, including consideration of availability, financial accessibility and acceptability of services, as these influence uptake of services. [11] Globally, there is strong qualitative evidence that people with disabilities experience difficulties in accessing healthcare, for instance, due to physical inaccessibility, information inaccessibility and costs.[4, 7] People with disabilities may also experience poorer quality services, for instance due to negative attitudes, lack of knowledge of health professionals or lack of accessible equipment. The full range of required services may not be available, as access to rehabilitation and specialist services is often poor in low and middle-income countries (LMICs). [12] Another concern is that on average, people with disabilities are poorer, [5] and face greater healthcare costs, which may become catastrophic and drive the family further into poverty.[4] As a result, people with disabilities face difficulties across the three domains of UHC: coverage, access to services needed, and at reasonable cost. The right to healthcare among people with disabilities is well established in international law and the United Nations Convention on the Rights of Persons with Disabilities (UNCRPD),[3] as well as in the laws of most countries. Yet, the limited literature available shows that there is often a large gap between policy/laws and practice.[4] [12, 13] Challenges to improving the focus on people with disabilities with respect to UHC has been the lack of data available, and the relatively simplistic tools used to measure access to health, thus far. [12, 13]

The aim of the current study is to describe access to healthcare services for people with disabilities within a national case-control study in Guatemala.[14] Guatemala is a middle-income country in Central America, with a population of 16.6 million people, with an estimated prevalence of disability in Guatemala of 10.2%.[14] In Guatemala, the Ministry of Public Health and Social Assistance operates a national network of clinical facilities to bring healthcare to the public. In many communities, the *centro de salud* (health centre) provides primary- and secondary-level healthcare services; smaller communities are often served through a *centro de convergencia* (convergence centre) or a *puesto de salud* (health post). Patients requiring tertiary-level healthcare services are referred to departmental or national hospitals. Private and non-profit institutions are also important healthcare providers, and overall the Guatemalan health system is characterised by high levels of fragmentation between these different actors. [15] Guatemala has ratified the UNCRPD, and national laws and policies are in place in Guatemala protecting the rights of people with disabilities to healthcare.

## Materials and methods

### Study design

A population-based case-control study was conducted in 2016 nested within a national survey of disability in Guatemala. [14] [16]

### Recruitment of cases and controls

Multi-stage stratified cluster random sampling with probability proportional to size procedures was used to identify a nationally representative sample, using the 2002 Census as the sampling frame. We randomly selected 56 clusters (enumeration areas) within each of the five regions in Guatemala. Within each cluster, we used compact segment sampling to divide the cluster into equal segments of approximately 50 people. One segment was randomly selected, and all households were visited door to door, until 50 people had been included.

Within each household, household-level socio-economic status (SES) indicators (e.g. assets, household characteristics) were collected as well as demographic data (age, sex, ethnicity, education, literacy and marital status) for all household members. Disability status was assessed for each household member aged 2+ years using the Washington Group Extended Set on Functioning for adults aged ≥18 years, and the UNICEF/Washington Group Extended Set on Functioning for children aged 2–17 years. [17] Disability was defined as people who reported “significant” functional limitations in at least one domain, namely:

- Adults:
  ○ Reporting “a lot of difficulty” or “cannot do” in seeing, hearing, walking, self-care, communication (understanding/being understood), cognition (remembering and concentrating), upper body (fine motor dexterity and upper body strength), and/or
  ○ Reporting “a lot” of anxiety/depression daily
- Children:
  ○ Aged 2–4: Reporting “a lot of difficulty” or “cannot do” in seeing, hearing, walking, fine motor dexterity, understanding, being understood, learning, playing and/or controlling behaviour
  ○ Aged 5–17: Reporting “a lot of difficulty” or “cannot do” in seeing, hearing, walking, self-care, understanding, being understood, learning, remembering, concentrating, accepting change, controlling behaviour, anxiety and/or depression

Questions were asked to an adult caregiver as a proxy for children under 10 years, or participants aged above 10 years who were unable to communicate independently.

All people with a disability (as defined above) were included in the nested case-control study. For each person identified as having a disability (“case”) one age and sex matched “control” who did not fulfil the case criteria was selected from within the same cluster. Controls were matched by age within +/-10 years for adults (aged 18+) and +/- 2 years for children (aged 2–17).

### Data collection

Informed written consent was sought from all participants in the case-control. Participants aged <18 years or for whom it was difficult to obtain consent directly (e.g. people with cognitive impairments) were asked for verbal assent, with written consent given by the caregiver/guardian, who remained throughout the interview. All cases and controls were interviewed using standardised questionnaires, including questions about healthcare access in a broad sense (the presence and treatment of specific health conditions, healthcare seeking behaviour, access to a range of rehabilitation services and assistive devices and perceptions of quality of services) (S1 Appendix). Interviewers were conducted in Spanish or in the dominant Mayan languages.

### Team, training and piloting

Five survey teams, two comprising three interviewers and three comprising four interviewers were recruited. Interviewers underwent a ten-day training on all aspects of the project protocol and methods.

Ethical approval for the study was provided by: the London School of Hygiene & Tropical Medicine (LSHTM) and the Comité de Ética Independiente en Investigación Latin Ethics, Guatemala. A National Directory of Disability Services was compiled with support from CBM (the NGO partner), Asociación de Asistencia Técnica y Capacitación en Educación y Discapacidad (ASCATED) and the National Council for the Care of Persons with Disabilities (CONADI), and distributed to the nearest public health service to each of the study clusters. We advised participants expressing desire for disability-related services to visit their nearest health service.

### Data analysis

All data were collected on android tablets using a bespoke mobile application and transferred daily to a secure, password-protected, cloud-based server. Data analysis was completed using STATA. We constructed a SES score using principal component analysis (PCA) of household asset ownership and household building materials. This SES score was then divided into quartiles. Multivariable logistic regression analysis was used to identify differences between people with and without disabilities in access to healthcare and rehabilitation services. These analyses were adjusted for age, sex, region and SES as potential confounding factors.

## Results

In total, 13,073 people were screened for disability (88% of those enumerated). The participants included were representative of the national population in terms of age and sex distribution. [16] The survey identified 707 people with disabilities, and 465 age- and sex matched controls without disabilities; It was not possible to identify an eligible control for each case due to the high prevalence of disability among older adults. Cases with disabilities were on average older than controls, but well-matched in terms of sex, regional distribution and SES (Table 1). The most common functional limitations among the cases were anxiety/depression (44%) followed by physical (31%) and visual (28%) difficulties.

**Table 1:** Socio-demographic characteristics of people with and without disabilities.

	People with disabilities (n = 707)	People without disabilities (n = 465)	Age, Sex, adjusted OR (95% CI)
	N (%)	N (%)	
**Age**			
5–14	95 (13%)	79 (17%)	0.4 (0.2–0.6)
15–24	96 (14%)	103 (22%)	0.3 (0.2–0.4)
25–54	266 (38%)	182 (39%)	0.5 (0.3–0.7)
55–64	80 (11%)	47 (10%)	0.5 (0.3–0.9)
65+	170 (24%)	54 (12%)	Baseline
**Sex**			
Male	253 (36%)	163 (35%)	Baseline
Female	454 (64%)	301 (65%)	1.1 (0.8–1.4)
**Region**			
Central	194 (27%)	123 (26%)	Baseline
North-East	66 (9%)	50 (11%)	0.7 (0.4–1.0)
North-West	233 (33%)	110 (24%)	1.3 (1.0–1.8)
South-East	55 (8%)	54 (12%)	0.5 (0.3–0.8)
South-West	159 (22%)	128 (28%)	0.7 (0.5–1.0)
**SES**			
1^st^ Quartile (poorest)	155 (22%)	116 (25%)	0.9 (0.6–1.2)
2^nd^ Quartile	198 (28%)	120 (26%)	1.1 (0.8–1.5)
3^rd^ Quartile	182 (26%)	118 (25%)	1.0 (0.7–1.4)
4^th^ Quartile (richest)	172 (24%)	111 (24%)	Baseline
**Functional limitation type**			
Seeing	200 (28%)	-	-
Hearing	104 (15%)	-	-
Physical	218 (31%)	-	-
Anxiety/Depression	314 (44%)	-	-
Self-care	80 (11%)	-	-
Cognition/Communication	69 (10%)	-	-

Cases were nearly three times more likely than controls to have reported a serious health problem in the past 12 months (aOR 2.8, 2.2–3.7) (Table 2). Among people with disabilities, reports of serious health problems was significantly more common among adults 18–49 (aOR 1.9, 1.2–2.9) and those aged 50+ (aOR 3.3, 2.1–5.1) compared to children. Furthermore, serious health problems were reportedly more common among people with significant limitations in physical (aOR 1.7, 1.2–2.5), anxiety/depression (aOR1.8, 1.3–2.5) or multiple domains (aOR 1.9, 1.3–2.7) compared to people with disabilities without that corresponding limitation. Most people reporting a serious health problem sought advice/treatment, both among people with and without disabilities (76% versus 72%, p>0.05), and they did not differ in type of facility where care was sought. People with disabilities were more likely to report that availability of services was a “big” problem (aOR 1.9, 1.4–2.6), being disrespected (aOR 1.9, 1.0–3.7) or finding it difficult to understand information given (aOR 1.6, 1.1–1.4) compared to controls.

**Table 2:** Healthcare seeking and experience among people with and without disabilities.

	People with disabilities N (%)	People without disabilities N (%)	Age, Sex, Region, SES adjusted OR (95% CI)
Serious health problem past 12 months	333 (47%)	105 (23%)	2.8 (2.2–3.7)
Sought advice/treatment	254 (76%)	78 (72%)	1.2 (0.7–2.1)
**Where sought advice/treatment**			
- Government Health Centre	52 (20%)	23 (28%)	Baseline
- Community Health Worker/ Health Post	10 (4%)	(6%)	1.1 (0.3–4.1)
- Government/IGSS Hospital	93 (36%)	23%)	1.9 (0.9–4.2)
- Pharmacy	24 (9%)	4 (5%)	2.2 (0.6–7.5)
- Private Clinic /Hospital	63 (25%)	35%)	0.8 (0.4–1.6)
- Traditional Healer/home remedy	12 (5%)	1 (1%)	-
Availability of health services			
- Never a problem	376 (53%)	301 (65%)	Baseline
- A little problem	117 (17%)	16%)	1.2 (0.8–1.7)
- A big problem	88 (19%)	88 (19%)	1.9 (1.4–2.6)
**Experience last time received healthcare**			
How did you feel?			
- Completely/mostly respected	435 (81%)	257 (85%)	Baseline
- Neither respected nor disrespected	56 (10%)	(10%)	1.1 (0.7–1.7)
- Completely/mostly disrespected	47 (9%)	13 (4%)	1.9 (1.0–3.7)*
Ease of understanding information			
- Easy	308 (57%)	196 (65%)	Baseline
- Neither easy nor difficult	109 (20%)	21%)	1.0 (0.7–1.4)
- Difficult	121 (22%)	42 (14%)	1.6 (1.1–1.4)*
Ease of being understood			
- Easy	323 (62%)	193 (64%)	Baseline
- Neither easy nor difficult	108 (20%)	21%)	0.9 (0.6–1.3)
- Difficult	106 (20%)	43 (14%)	1.3 (0.8–1.9)*

*excluding those who have never previously sought healthcare, p<0.05

Table 3 shows coverage of treatment for 17 general health conditions, where there was a report of doctor-diagnosed condition, comparing people with and without disabilities. Most health conditions were reported to be more common among people with disabilities, although for some conditions the numbers of people affected were small and the associations were not always statistically significant. Overall, people with disabilities were almost three times more likely to have any of the listed conditions compared to those without disabilities (aOR 2.9, 2.2–3.8). Across the individual conditions, there were no clear patterns as to whether people with disabilities were more or less likely to be receiving treatment, although numbers were often too small to allow meaningful comparison. Overall, people with disabilities were more likely to be receiving treatment if they had a diagnosed condition compared to controls (aOR 1.4, 1.0–1.9).

**Table 3:** Coverage of treatment for general health conditions, comparing people with and without disabilities.

	Told they have condition by doctor			Receiving treatment, if have condition		
	People with disabilities	People without disabilities	Age, Sex, Region, SES adjusted	People with disabilities	People without disabilities	Age, Sex, Region, SES adjusted
	N (%)	N (%)	OR (95% CI)	N (%)	N (%)	OR (95% CI)
Diabetes	63 (9%)	26 (6%)	1.3 (0.8–2.1)	48 (76%)*	15 (57%)	2.1 (0.7–6.6)
Hypertension	164 (23%)	55 (12%)	1.3 (0.8–2.1)	59 (36%)*	18 (33%)	1.0 (0.5–2.0)
Heart disease, Coronary Disease, Heart Attack	126 (18%)	33 (7%)	2.6 (1.7–3.9)	46 (36%)	8 (21%)	1.2 (0.5–3.2)
Chronic Bronchitis or Emphysema	70 (10%)	18 (4%)	2.3 (1.3–4.0)	45 (63%)	0.6 (77%)	0.6 (0.2–2.6)
Asthma, allergic respiratory disease	68 (10%)	24 (5%)	1.8 (1.1–2.9)	33 (49%)	10 (42%)	1.1 (0.3–3.5)
Migraine	289 (41%)	105 (23%)	2.4 (1.8–3.2)	120 (41%)	39 (37%)	1.3 (0.8–2.2)
Stroke	53 (8%)	9 (2%)	3.6 (1.7–7.6)	26 (47%)	5 (39%)	0.4 (0.1–2.3)
Tumour or cancer	15 (2%)	1 (0.2%)	-	11 (65%)	1 (20%)	-
Kidney diseases	61 (9%)	13 (3%)	3.2 (1.7–6.1)	34 (56%)	6 (46%)	3.0 (0.5–17.7)
Skin diseases	83 (12%)	31 (7%)	1.8 (1.1–2.8)	32 (39%)	10 (32%)	1.5 (0.5–3.9)
Tuberculosis	4 (0.6%)	2 (0.4%)	-	2 (33%)	1 (17%)	-
Sleep problems	278 (39%)	84 (18%)	2.5 (1.9–3.4)	55 (20%)	15 (18%)	1.1 (0.6–2.1)
Tinnitus	188 (27%)	84 (18%)	1.4 (1.1–2.0)	18 (10%)	5 (6%)	1.8 (0.6–5.4)
Severe diarrhea	89 (13%)	31 (7%)	1.8 (1.2–2.8)	54 (61%)	16 (51%)	0.7 (0.2–2.1)
Perinatal complications	16 (4%)	10 (4%)	1.3 (0.6–3.0)	4 (25%)	5 (50%)	0.5 (0.1–3.1)
Malnutrition	68 (10%)	16 (3%)	2.9 (1.7–5.2)	24 (34%)	6 (30%)	0.3 (0.1–1.4)
Mosquito borne illness	138 (20%)	80 (17%)	1.2 (0.9–1.7)	98 (70%)	66 (79%)	1.2 (0.6–2.2)
Any of above conditions	583 (83%)	271 (60%)	2.9 (2.2–3.8)	357 (61%)	149 (53%)	1.4 (1.0–1.9)

*Treatment through medication

Antenatal care coverage was assessed for women aged 15–49 years who had given birth in the last 5 years. Women with disabilities were significantly less likely to seek antenatal care (aOR 0.4, 0.1–1.0) compared to controls, but were more likely to deliver at a health centre/hospital rather than at home (aOR 4.0, 1.4–11.6) and to have delivery assisted by a doctor/nurse rather than a non-medical professional (aOR 2.9, 1.0–8.2). Vaccination coverage among children aged 5–9 was high overall, both among children with disabilities (94%) and those without (88%, aOR 2.6, 0.3–20.2).

Unsurprisingly, people with disabilities were more likely to have been told that they had one of a list of impairment-related health conditions in comparison to controls (overall aOR 5.3, 4.0–7.1) (Table 4). Coverage of treatment was consistently low (≤33%), even among this group who had received a doctor-diagnosis for the condition. Few people with disabilities reported needing medical rehabilitation, specialist health services, or assistive devices (with the exception of glasses), although in reality most people with disabilities would benefit from at least one of these services (Table 5). Most people who reported needing medical rehabilitation or specialist care had received these services. However, among those who reported having needed assistive devices, few had ever received these.

**Table 4:** Coverage of treatment for impairment-related health conditions, comparing people with and without disabilities.

	Told they have condition by doctor			Receiving treatment, if have condition		
	People with disabilities	People without disabilities	Age, Sex, Region, SES adjusted	People with disabilities	People without disabilities	Age, Sex, Region, SES adjusted
	N (%)	N (%)	OR (95% CI)	N (%)	N (%)	OR (95% CI)
Vision loss	333 (47%)	90 (19%)	3.2 (2.4–4.3)	52 (16%)	15 (16%)	1.0 (0.5–2.0)
Hearing loss	189 (27%)	33 (7%)	4.0 (2.6–6.0)	15 (8%)	4 (11%)	0.6 (0.1–2.1)
Arthritis, arthosis	173 (25%)	51 (10%)	2.1 (1.5–3.0)	57 (33%)	21 (39%)	0.5 (0.3–1.1)
Back pain or disc problems	265 (38%)	92 (20%)	2.0 (1.5–2.7)	63 (24%)	27 (29%)	0.7 (0.4–1.3)
Depression or Anxiety	292 (41%)	76 (16%)	3.7 (2.8–5.1)	40 (13%)	17 (21%)	0.6 (0.3–1.2)
Dementia	37 (5%)	4 (1%)	6.2 (2.2–18.0)	1 (3%)	0 (0%)	-
Mental (psychiatric) or behavioural disorders	33 (5%)	8 (2%)	3.3 (1.5–7.4)	3 (9%)	0 (0%)	-
Any of above conditions	573 (81%)	202 (43%)	5.3 (4.0–7.1)	177 (31%)	67 (33%)	0.9 (0.6–1.3)

**Table 5:** Awareness and access to rehabilitation services among people with disabilities.

	Have needed services	Have received services	
	N	N	%
Medical Rehabilitation	39	24	62%
Specialist Health Services	40	28	70%
**Assistive devices**			
Glasses	361	81	22%
Magnifying Glass	145	16	11%
Braille	10	1	10%
White Cane	58	19	33%
Hearing Aid	89	3	3%
Wheelchair	62	23	37%
Crutches	29	9	31%
Walking Stick	77	37	48%
Guide (another person)	30	11	37%
Standing Frame	32	8	25%
Prosthesis	13	6	46%

## Discussion

This large national case-control study from Guatemala found that people with disabilities were approximately three times more likely to report a serious health condition or a diagnosis of a specific general health condition, compared to people without disabilities. General healthcare treatment coverage did not appear to be worse among people with than without disabilities. In contrast, women with disabilities were less likely to seek antenatal care. Concerns were raised about quality of care received for people with disabilities, as they were more likely to report being disrespected or finding it difficult to understand information given. Coverage of treatment for impairment-related health conditions was low, as was awareness of and access to rehabilitation services and assistive devices.

Our results are consistent with the general literature that shows that on average people with disabilities experience worse health than those without disabilities [8, 18–21]. There are different explanations for this association and the directions of causation between disability and ill health are complex and non- linear. The health condition underlying the person with disabilities’s impairment, or the impairment itself, may produce further health consequences (e.g. spinal cord injury increases vulnerability to pressure sores, and urinary tract infections). Disability may also be linked to poor health through other pathways. Disability is more common in older people, who are also most vulnerable to poor health, and adjustment for age may not have entirely removed this confounding. Access to preventive and curative interventions may be worse for people with disabilities, so that health conditions are more prevalent or severe. [4][12, 13] Finally, people with disabilities often have a disadvantaged and marginalised structural position in society, [5] which is linked to poor health.

As people with disabilities are more likely to experience poor health they will have higher healthcare needs. Our findings support previous studies including the World Health Surveys, conducted across 51 countries, where people with disabilities were consistently more likely to seek inpatient or outpatient care. [4, 22, 23] Higher utilization does not, however, equate to equity in coverage (i.e. receipt of care when needed), and few studies have assessed whether coverage of health services varies between people with and without disabilities. [13] The World Health Surveys showed that people with disabilities were more likely to report needing services but not using them, indicating lower coverage. [4] In contrast, other studies have not found differences in seeking care after “serious illness” between people with and without disabilities, [8, 9, 24] but this may not reflect the experience of coverage for more routine non-urgent healthcare. The current study finds that coverage of medical treatment across a range of conditions is higher for people with disabilities compared to those without. This finding is surprising, and needs further exploration. Coverage of specialist services and rehabilitation was, however, low in this study echoing findings from the general literature. [4][12] Poor coverage of these services shows a large gap in the health system for people with disabilities, and highlights that UHC will be difficult to achieve without improving the provision of the whole range of services needed by people with disabilities. Potential explanations for the low rehabilitation coverage is lack of availability or access (particularly for those in rural areas), lack of knowledge or referrals and costs.

Even if people with disabilities do not experience lower coverage of healthcare services, they may have to overcome more hurdles in order to obtain care. The qualitative literature is rich with examples of how people with disabilities face difficulties in accessing healthcare with barriers ranging from physical inaccessibility of facilities, difficulties with communication, stigma, financial barriers, and inadequate training and facilities at healthcare services [25–28]. Furthermore, people with disabilities also report that the quality of care that they receive is often not appropriate and that they face negative and discriminatory attitudes and lack of recognition of their particular needs. [25, 26, 28]. The current study echoed these concerns about quality of healthcare provided to people with disabilities.

There are strengths and limitations of this study that should be considered when evaluating the findings. This was a large study, conducted across the whole of Guatemala, and drawing a nationally representative sample. Data were collected on diagnosis of a range of health conditions, both general and impairment-specific, as well as access to related services and perceived quality of services. However, no measures were made of affordability of healthcare, despite evidence showing that people with disabilities pay more for accessing healthcare services. [13, 25, 28] Furthermore, the presence of health conditions and treatment was self-reported, rather than clinically verified, small numbers prohibited comparisons in some cases (e.g. vaccination coverage) and controls were not identified for all cases.

Issues of poor health among people with disabilities are important to address, since it leads to reduced functioning, social participation and quality of life and increased poverty, suffering, further morbidity and early mortality. [29, 30] This study provides evidence of the need to improve inclusion of people with disabilities in the health system in Guatemala, particularly with respect to improving coverage of specialist services and strengthening the quality of general health services provided. These efforts should be an important priority, since disability is experienced by one in ten people in Guatemala, [14] and providing good access to health for people with disabilities will ensure that their rights, as well as needs, are met. [3] Changes in the health system to make them inclusive, barrier free and holistic are likely to improve access for other vulnerable groups (e.g. older people, minority language speakers), as well as the general population. Improving availability of rehabilitation and specialist services will also benefit people with short-term impairments, such as those experiencing trauma or stroke. [31] Overall, these changes will help in achievement of UHC–by ensuring that healthcare services reach the whole population, with the full range of services, including rehabilitation and assistive devices.

In conclusion, people with disabilities in Guatemala are a large group who experience greater need for healthcare, but face difficulties in accessing the full range of services needed. It may therefore be difficult to achieve UHC without considering this group, however, evidence or guidance is lacking on how to provide more inclusive healthcare services.[31, 32] Notably, a recent overview of systematic reviews showed very limited data addressing disability within delivery, financial or governance arrangements for health systems in low-income countries, or for implementation strategies.[33–36] More evidence is needed taking a health system approach to addressing the issues and identifying solutions, and this must include the development of tools to measure access in the broader sense, including improving quality of care and affordability, as well as geographic access. [37]

## Supporting information

S1 Appendix Guatemala national disability survey case control questionnaire. (PDF)
