# Supplementary material for: Can we achieve universal health coverage without a focus on disability? Results from a national case-control study in Guatemala
Source: PLoS One. 2018 Dec 27;13(12):e0209774. doi: 10.1371/journal.pone.0209774 (PMC6307777; doi:10.1371/journal.pone.0209774)
Supplement: S1 Appendix — (PDF) [file pone.0209774.s001.pdf]

Guatemala National Disability Survey Case Control Questionnaire

**COVERSHEET**		
1	Interviewer ID No.	☐☐ ✍
2	Region No.	☐☐ ✍
3	Cluster No.	☐☐☐ ✍
4	House No.	☐☐ ✍
5	Participant Line No. from roster	☐☐ ✍
6	Participant ID No.	☐☐ ✍
7	Participant Name	✍
8	Participant Informed Consent No.	☐☐☐☐ ✍
9	Date (Day/Month/Year)	/ / ✍
10	Language of Interview	Spanish O (1)<br>Mam O (2)<br>K'iche O (3)<br>Cakchiquel O (4)<br>K'ekchi' O (5)<br>Other O (6)
10.1	Specify if other	✍
11	Case or Control<br>*Enter this response carefully!*	Case (Person with a disability) O (1)<br>Control O (2)
12	What is the participant's age (years)?<br>*Enter this response carefully!*	☐☐☐ ✍
13	What is the participant's gender (report)?<br>*Enter this response carefully!*	Male O (1)<br>Female O (2)<br>Other O (3)<br>Refuse response O (4)
13.1	Specify if other	✍
14	Person interviewed	Direct interview with case/control O (1)<br>Interview with proxy only O (2)<br>Interview with proxy and case/control together O (3)<br>Interview via formal translator O (4)<br>Interview via informal community translator O (5)
15	Line number of proxy respondent	☐☐ ✍
**ANTHROPOMETRY**		
16.1	Middle Upper Arm Circumference	MUAC Measured O (1)<br>MUAC not Measured O (2)
16.2	Specify Reason if not measured	✍
16.3	MUAC Measurement in Centimeters	1st
16.4		2nd
17.1	Tibia Length	Tibia Length Measured O (1)<br>Tibia Length not Measured O (2)
17.2	Specify Reason if not measured	✍

Guatemala National Disability Survey Case Control Questionnaire		
17.3	Tibia Measurement in Centimeters	1st
17.4		2nd
18.1	Weight Measurement	Weight Measured O (1)<br>Weight not measured – wheelchair user O (2)<br>Weight not measured – Other reason O (3)
18.2	Specify if other	✍
18.3	Weight in kilograms	1st
18.4		2nd
19.1	Height Measurement	Height Measured standing O (1)<br>Height measured lying down O (2)<br>Height not measured O (3)
19.2	Reason if height not measured	✍
19.3	Height in Centimetres	st
19.4		2nd
**WATER AND SANITATION QUESTIONS**		
1	What kind of toilet facilities do members of your household usually use?	**Flush/pour flush to:**<br><br>Piped sewer system O (1)<br>Septic tank O (2)<br>Pit Latrine O (3)<br>Elsewhere O (4)<br>Unknown place/not sure O (5)<br><br>**Non flush/pour to:**<br>Ventilated improved pit latrine O (6)<br>Pit Latrine with slab O (7)<br>Pit latrine without slab/ open pit O (8)<br>Composting toilet O (9)<br>Bucket O (10)<br>hanging latrine O (11)<br>No facilities or bush or field O (12)<br>Other (Specify) O (13)
1.2	Specify if other	✍
2	Do you share this facility with other households?	Used only by your household O (1)<br>Shared with other households O (2)<br>Public/ Communal/ Community Latrine O (3)
3	Do you use the same toilet facility as other members of your household?	No O (0)<br>If yes go to Q5 Yes O (1)

Guatemala National Disability Survey Case Control Questionnaire		
4	If no, why do you use a different toilet facility from other members of your household? (main reason)	It would be physically impossible O (1)<br>I'm not allowed/ others would not like it O (2)<br>I might face verbal or physical abuse O (3)<br>I would be embarrassed O (4)<br>Other O (5)
4.1	Specify if other	✍
5	What kind of toilet facility do you usually use	Flush Toilet O (1)<br>Traditional Latrine O (2)<br>Ventilation Improved Pit Latrine O (3)<br>Bowl/Bucket O (4)<br>No toilet (5)<br>Other Specify (6)
5.1	Specify if other	✍
6	Are you usually able to use the toilet facility without you or your clothes coming into contact with faeces?	No O (0)<br>Yes O (1)
7	Are you able to use this toilet facility without assistance from another person?	No O (0)<br>Yes O (1)
8	Do you make any changes to your daily routine or practices in your use of the toilet?<br><br>(*tick all that apply*)	I restrict my use of the toilet O (1)<br>I limit my food intake O (2)<br>I limit my fluid intake O (3)<br>I sometimes soil myself O (4)<br>I use special clothing/pads/diaper O (5)<br>I store a bowl/ bedpan nearby for use O (6)<br>Other (Specify) O (7)
8.1	Specify if other	✍
9	What is the main source of drinking water for members of your household?	Piped into dwelling O (1)<br>Piped into compound, yard or plot O (2)<br>Piped into neighbour's compound O (3)<br>Public tap/standpipe O (4)<br>Tubewell/Borehole O (5)<br>Protected Well O (6)<br>Unprotected Well O (7)<br>Protected spring O (8)<br>Unprotected spring O (9)<br>Rainwater collection O (10)<br>Tanker Truck O (11)<br>cart with small tank/drum O (12)<br>Surface water O (13)<br>Bottled Water O (14)<br>Other (specify) O (15)
9.1	Specify if other	✍

Guatemala National Disability Survey Case Control Questionnaire		
10	How long does it take to go there, get water and come back (minutes) *nb "00" if piped into dwelling*	☐☐☐ ✍
11	Are you able to access drinking water when you need it?	No O (0)<br>If yes go to next section Yes O (1)
12	If no, why are you not able to access drinking water when you need it?	I do not have the physical strength eg. The container is too heavy to lift O (1)<br>I am visually impaired/ blind O (2)<br>I have mobility difficulties and cannot grasp/hold or balance the container O (3)<br>The container is out of reach O (4)<br>I am not allowed/ discouraged to access water O (5)<br>There is no-one to assist me O (6)<br>Other (specify) O (7)
12.1	Specify if other	✍
**LITERACY (16+)**		
1	Can you read well, a little or not at all?	Well O (1)<br>A little (2)<br>Not at all (3)
2	Have you ever attended school?	No O (0)<br>IF YES GO TO LIVELIHOODS SECTION Yes O (1)
3	What is the main reason why you did not receive formal education?	No school O (1)<br>Lack of money O (2)<br>Needed to work O (3)<br>Education not very useful O (4)<br>Being disabled was refused O (5)<br>Don't like school O (6)<br>Too much household work O (7)<br>Family does not allow it O (8)<br>No Transport O (9)<br>Other O (10)
3.1	Specify if other	✍
**EDUCATION (<18)**		
1	Are you currently enrolled in school?	IF NO, go to Q7 No O (0)<br>Yes O (1)
2	Are you enrolled in the same grade as other children your age?	Yes O (1)<br>No, lower grade than other children my age O (2)<br>No, a higher grade than other children my age O (3)

Guatemala National Disability Survey Case Control Questionnaire		
3	Is the school you are in a mainstream/regular school or special school?	Mainstream / regular O (1)<br>Special School (2)<br>Integrated O (3)<br>Mainstream with special/extra classes O (4)<br>Other (5)
3.1	Specify if other	✍
4	In the last month of school, how many days did you miss?	☐☐ ✍
5	Have you ever repeated a grade at school?	IF NO, go to Q11 No O (0)<br>Yes O (1)
6	If yes, how many times have you repeated a grade at school?	☐☐<br>Go to Q11 ✍
7	If not currently enrolled, have you ever attended school?	IF NO, go to Q10 No O (0)<br>Yes O (1)
8	What is the highest level of education you completed?	Primary O (1)<br>Secondary O (2)<br>High School O (3)<br>University O (4)
9	What was the highest grade that you completed?	☐☐ ✍
10	If you have never attended or are currently not attending school, what is the main reason?	Not enough money O (1)<br>Lack of interest to go to school O (2)<br>Lack of school nearby O (3)<br>Nearby school is not accessible O (4)<br>Illness (<1 month) O (5)<br>Illness (>1 month) O (6)<br>Attendance refused by school O (7)<br>Negative attitudes of other students O (8)<br>Negative attitudes of other teachers O (9)<br>Lack of accessible resources to assist child O (10)<br>Child works O (11)<br>Other O (12)
10.1	Specify if other	✍
11	At school are you able to use the same toilet facility as other pupils?	No O (0)<br>IF YES, go to Q13 Yes O (1)
12	If no, what is the main reason why not?	It would be physically impossible O (1)<br>I could not use it without getting myself or my clothes soiled O (2)<br>I'm not allowed/ others would not like it O (3)<br>I might face verbal or physical abuse O (4)<br>I would be embarrassed O (5)<br>Other (6)

Guatemala National Disability Survey Case Control Questionnaire		
12.1	Specify if other	✍
13	At school are you able to access drinking water from the same source as other pupils?	No O (0)<br>IF YES, go to Q15 Yes O (1)
14	If no, what is the main reason why not?	It would be physically impossible O (1)<br>I'm not allowed/ others would not like it O (2)<br>I might face verbal or physical abuse O (3)<br>I would be embarrassed O (4)<br>My clothes would get dirty O (5)<br>Other (specify) (6)
14.1	Specify if other	✍
15	At school are you able to wash your hands at the same place as other pupils?	No O (0)<br>IF YES, go to next section Yes O (1)
16	If no, what is the main reason why not?	It would be physically impossible O (1)<br>I'm not allowed/<br>others would not like it O (2)<br>I might face verbal or physical abuse O (3)<br>I would be embarrassed O (4)<br>My clothes would get dirty (5)<br>Other (specify) (6)
16.1	Specify if other	✍
**FAMILY DYNAMICS (Aged <18)**		
1	Does your father live with you?	IF NO, go to next Q3 No O (0)<br>Yes O (1)
2	If yes, is he your biological father?	No O (0)<br>IF YES, go to next Q4 Yes O (1)<br>Don't know O (2)
3	Where does your biological father live?	Working away from the household O (1)<br>Has other famiy/ does not live in household O (2)<br>(NEXT SECTION) Father dead O (88)<br>Not known O (99)
4	In the last 6 months, how often have you seen your biological father?	Daily O (1)<br>Weekly O (2)<br>Monthly O (3)<br>Less than monthly O (4)<br>Never in the last 6 months O (5)
**LIVELIHOODS (AGED 18+)**		
1	Other than domestic work in the household have you done any work in the last seven days?	No O (0)<br>IF YES, go to Q4 Yes O (1)
2	Although you did not work in the last seven days, do you have any job or business from which you were absent for leave, illness, vacation, or any other such reason?	No O (0)<br>IF YES, go to Q4 Yes O (1)
3	Have you done any work in the last 12 months?	IF NO, go to Q9 No O (0)<br>Yes O (1)

Guatemala National Disability Survey Case Control Questionnaire		
4	What is your occupation status?	Working For Wages Or Salary With An Employer (Full- Or Part-Time) O (1)<br>Working For Wages, But Currently On Sick Leave For More Than Three Months O (2)<br>Being Self-Employed (subsistence) O (3)<br>Being Self-Employed (employs others) O (4)<br>Working As Unpaid Family Member (E.G. Working In Family Business) O (5)<br>Is Available And Actively Looking For Work O (6)<br>Is Engaged In Training O (7)<br>Is Engaged In Home Duties (Including Child Care) O (8)<br>Is Not Working Or Seeking Work Because Of A Health Condition Or Disability O (9)<br>Is Not Working Or Seeking Work For Other Reasons O (10)<br>Is Retired O (11)<br>Other (specify) (12)
4.1	Specify if other	✍
5	Who do you work for?	Work on your own/household's business O (1)<br>work for someone who is not a member of your household O (2)<br>work on farm owned by yourself or household member O (3)<br>Work on farm rented by yourself or household member O (4)<br>Other (specify) (5)
5.1	Specify if other	✍
6	What is your primary work activity?	Agriculture – field (1)<br>Agriculture – in-home (2)<br>Livestock raising (3)<br>Poultry raising (4)<br>Artesenal crafts (5)<br>Sales/commercial activity (6)<br>Professional activity (7)<br>Other (8)
6.1	Specify if other	✍
7	Do you usually work throughout the year, or do you work seasonally, or only once in a while?	Throughout the year O (1)<br>Seasonally/ part of the year O (2)<br>Once in a while O (3)
8	Are you paid in money (cash or bank transfer) or kind for this work, or are you not paid at all?	Money only O (1)<br>Money and in kind O (2)<br>In Kind only O (3)<br>Not paid O (4)<br>GO TO Q10

Guatemala National Disability Survey Case Control Questionnaire		
9	If not working, what is the main reason?	Student O (1)<br>Childcare/ household duties O (2)<br>Too old/retired O (3)<br>My physical health does not allow me to O (4)<br>My mental health does not allow me to O (5)<br>No one will give job because of disability O (6)<br>Long illness (> 1 month) O (7)<br>I am looking for my first job O (8)<br>No job opportunities in the area O (9)<br>Quit job O (10)<br>Suspended from job O (11)<br>Other (specify) O (12)
9.1	Specify if other	✍
	Do you receive any of the following benefits from the state?	
10	Retirement pension	No O (0)<br>Yes, from IGSS (social security)O (1)<br>Yes, from government (2)
11	Disability pension	No O (0)<br>Yes, from IGSS (social security) O (1)<br>Yes, from government (2)
12	Family Allowance	No O (0)<br>Yes O (1)
13	Other	No O (0)<br>Yes O (1)
14	Specify if other	✍
	Are you involved in any of the following non-state activities?	
15	Social security benefits	No O (0)<br>Yes O (1)
16	Microfinance	No O (0)<br>Yes O (1)
17	Cash for work schemes	No O (0)<br>Yes O (1)
18	Asset Schemes	No O (0)<br>Yes O (1)
19	Remittances	No O (0)<br>Yes O (1)
20	Other	No O (0)<br>Yes O (1)
21	Specify if other	✍

QUALITY OF LIFE (Age 10+)		
	The following questions ask how you feel about your quality of life. I will read out each question to you, along with the response options. Please choose the answer that appears most appropriate. If you are unsure about which response to give to a question, the first response you think of is often the best one<br>*Please keep in mind your standards, hopes, pleasures and concerns. We ask that you think about your life in the last four weeks (The overall quality of life and general health facet).*	
1	How would you rate your quality of life?	Very Poor O (1)<br>Poor O (2)<br>Neither Poor nor good O (3)<br>Good O (4)<br>Very Good O (5)
2	2. How satisfied are you with your health?	Very dissatisfied O (1)<br>Dissatisfied O (2)<br>Neither satisfied nor disaatisfied O (3)<br>Satisfied O (4)<br>Very Satisfied O (5)
	The following questions ask about how much you have experienced certain things in the last four weeks.	
3	To what extent do you feel that physical pain prevents you from doing what you need to do?	Not at all O (5)<br>A little O (4)<br>A moderate amount O (3)<br>Very much O (2)<br>An extreme amount O (1)
4	How much do you need any medical treatment to function in your daily life?	Not at all O (5)<br>A little O (4)<br>A moderate amount O (3)<br>Very much O (2)<br>An extreme amount O (1)
5	How much do you enjoy life?	Not at all O (1)<br>A little O (2)<br>A moderate amount O (3)<br>Very much O (4)<br>An extreme amount O (5)
6	To what extent do you feel your life to be meaningful?	Not at all O (1)<br>A little O (2)<br>A moderate amount O (3)<br>Very much O (4)<br>An extreme amount O (5)
7	How well are you able to concentrate?	Not at all O (1)<br>A little O (2)<br>A moderate amount O (3)<br>Very much O (4)<br>Extremely O (5)
8	How safe do you feel in your daily life?	Not at all O (1)<br>A little O (2)<br>A moderate amount O (3)<br>Very much O (4)<br>Extremely O (5)

Guatemala National Disability Survey Case Control Questionnaire		
9	How healthy is your physical environment?	Not at all O (1)<br>A little O (2)<br>A moderate amount O (3)<br>Very much O (4)<br>Extremely O (5)
10	Do you have enough energy for everyday life?	Not at all O (1)<br>A little O (2)<br>Moderately O (3)<br>Mostly O (4)<br>Completely O (5)
11	Are you able to accept your bodily appearance?	Not at all O (1)<br>A little O (2)<br>Moderately O (3)<br>Mostly O (4)<br>Completely O (5)
	The following questions ask about how completely you experience or were able to do certain things in the last four weeks	
12	Have you enough money to meet your needs?	Not at all O (1)<br>A little O (2)<br>Moderately O (3)<br>Mostly O (4)<br>Completely O (5)
13	How available to you is the information that you need in your day-to-day life?	Not at all O (1)<br>A little O (2)<br>Moderately O (3)<br>Mostly O (4)<br>Completely O (5)
14	To what extent do you have the opportunity for leisure activities?	Not at all O (1)<br>A little O (2)<br>Moderately O (3)<br>Mostly O (4)<br>Completely O (5)
15	How well are you able to get around?	Very poor O (1)<br>Poor O (2)<br>Neither poor nor good O (3)<br>Good O (4)<br>Very good O (5)
16	How satisfied are you with your sleep?	Very dissatisfied O (1)<br>Dissatisfied O (2)<br>Neither satisfied nor disaatisfied O (3)<br>Satisfied O (4)<br>Very Satisfied O (5)
17	How satisfied are you with your ability to perform your daily living activities?	Very dissatisfied O (1)<br>Dissatisfied O (2)<br>Neither satisfied nor disaatisfied O (3)<br>Satisfied O (4)<br>Very Satisfied O (5)

Guatemala National Disability Survey Case Control Questionnaire		
18	How satisfied are you with your capacity for work?	Very dissatisfied O (1)<br>Dissatisfied O (2)<br>Neither satisfied nor disaatisfied O (3)<br>Satisfied O (4)<br>Very Satisfied O (5)
19	How satisfied are you with yourself?	Very dissatisfied O (1)<br>Dissatisfied O (2)<br>Neither satisfied nor disaatisfied O (3)<br>Satisfied O (4)<br>Very Satisfied O (5)
20	How satisfied are you with your personal relationships?	Very dissatisfied O (1)<br>Dissatisfied O (2)<br>Neither satisfied nor disaatisfied O (3)<br>Satisfied O (4)<br>Very Satisfied O (5)
21	How satisfied are you with your sex life?<br><br>*Age 10-17 replace this question with "How satisfied are you with the respect you receive from others?"*	Very dissatisfied O (1)<br>Dissatisfied O (2)<br>Neither satisfied nor disaatisfied O (3)<br>Satisfied O (4)<br>Very Satisfied O (5)
22	How satisfied are you with the support you get from your friends?	Very dissatisfied O (1)<br>Dissatisfied O (2)<br>Neither satisfied nor disaatisfied O (3)<br>Satisfied O (4)<br>Very Satisfied O (5)
23	How satisfied are you with the conditions of your living place?	Very dissatisfied O (1)<br>Dissatisfied O (2)<br>Neither satisfied nor disaatisfied O (3)<br>Satisfied O (4)<br>Very Satisfied O (5)
24	How satisfied are you with your access to health services?	Very dissatisfied O (1)<br>Dissatisfied O (2)<br>Neither satisfied nor disaatisfied O (3)<br>Satisfied O (4)<br>Very Satisfied O (5)
25	How satisfied are you with your transport?	Very dissatisfied O (1)<br>Dissatisfied O (2)<br>Neither satisfied nor disaatisfied O (3)<br>Satisfied O (4)<br>Very Satisfied O (5)
	The following question refers to how often you have felt or experienced certain things in the last four weeks	
26	How often do you have negative feelings such as blue mood, despair, anxiety, depression?	Never O (5)<br>Seldom O (4)<br>Quite often O (3)<br>Very Often O (2)<br>Always O (1)

Guatemala National Disability Survey Case Control Questionnaire		
**HEALTH AND ANTENATAL**		
	For cases only:	
1	What do you think is the main cause of your functional limitation?	From birth O (1)<br>Trauma O (2)<br>Violence O (3)<br>Illness O (4)<br>Ageing O (5)<br>War O (6)<br>Other O (7)
2	Specify if other	✍
3	How old where you when you first experienced functional limitation?<br>000 = from birth<br>199 = Don't know/refused	☐☐☐ ✍
	For all participants	
4	Have you had any serious health problems during the last 12 months	IF NO GO TO Q9 No O (0)<br>Yes O (1)
5	If yes, what type of serious health event(s) or problem(s) did you experience during this period? (tick all that apply)	Severe Diarrhea O (1)<br>Acute respiratory tract infection/ pneumonia O (2)<br>Malaria O (3)<br>Dengue O (4)<br>Zika O (5)<br>Chikungunya O (6)<br>eye problems O (7)<br>Malnutrition O (8)<br>Vaccine-preventable diseases O (9)<br>Chronic Illness O (10)<br>Accident/ Illness O (11)<br>Don't know/<br>no information provided O (12)<br>Other O (13)
5.1	Specify if other	✍
6	Did you seek advice or treatment for any of these problems?	GO TO Q9 No O (0)<br>Yes O (1)
7	Where did you seek advice or treatment for the most recent of these serious health events or problems?<br><br>GO TO QUESTION 9	Community Health Worker/ Government Health Post O (1)<br>Government Health Centre O (2)<br>Government Hospital O (3)<br>IGSS supported hospital O (4)<br>Pharmacy O (5)<br>Non Governmental Organization/Mobile Clinic O (6)<br>Private Clinic O (7)<br>Private Hospital O (8)<br>Traditional Healer O (9)<br>Other (10)
7.1	Specify if other	✍
8	Why did you not seek advice or treatment?	✍

Guatemala National Disability Survey Case Control Questionnaire

9	Thinking about the last time that you sought advice or treatment for a serious health condition, how respected did you feel?	Completely respected O (1)<br>Mostly respected O (2)<br>Neither respected nor disrespected O (3)<br>Mostly disrespected O (4)<br>Completely disrespected O (5)<br>NA – has never received advice or treatment for a serious health condition O (6)			
10	Thinking about the last time that you sought advice or treatment for a serious health condition, how easy was it for you to understand the information given to you?	Easy O (1)<br>Neither easy nor difficult O (2)<br>Difficult O (3)<br>NA – has never received advice or treatment for a serious health condition O (4)			
11	Thinking about the last time that you sought advice or treatment for a serious health condition, how easy was it for you to be understood by the health provider?	Easy O (1)<br>Neither easy nor difficult O (2)<br>Difficult O (3)<br>NA – has never received advice or treatment for a serious health condition O (4)			
	Repeat for each of the following health conditions:				
		x.1 Do you have this condtion?<br>No (0)<br>Yes (1)<br><br>IF NO GO TO NEXT Q	x.2 Have you ever been told by a doctor that you have this condition?<br>No (0)<br>Yes (1)	x.3 In the last 12 months have you received any medication for this condition?<br>No (0)<br>Yes (1)	x.4 In the last 12 months have you been given any other treatment for this condition?<br>No (0)<br>Yes (1)
12	Vision loss				
13	Hearing Loss				
14	Arthritis, arthrosis				
15	Heart disease, Coronary Disease, Heart Attack				
16	Chronic Bronchitis or Emphysema				
17	Asthma, allergic respiratory disease				
18	Back pain or disc problems				
19	Migraine (recurrent headaches)				
20	Stroke e.g. cerebral bleeding				
21	Depression or Anxiety				
22	Tumour or cancer (including blood cancer)				
23	Dementia				
24	Kidney diseases				
25	Skin diseases e.g. Psoriasis				
26	Tuberculosis				
27	Mental (psychiatric) or behavioural disorders				
28	Sleep problems				
29	Tinnitus (ringing, roaring, or buzzing in your ears that lasts for 5 minutes or longer over the last 12 months)				

30	Severe Diarrhea				
31	Perinatal complications				
32	Malnutrition				
33	Mosquito borne illness (dengue, malaria, chikungunya, zika)				

34	Have you ever heard of an illness called diabetes, or “problems with blood sugar”?	IF NO GO TO QU 39 No O (0)<br>Yes O (1)
35	Have you ever had a check with a health professional to see if you have diabetes (have you ever had blood sugar tests)?	No O (0)<br>Yes O (1)<br>Don’t know O (2)
36	Have you ever been told by a doctor or other health worker that you have high blood sugar or diabetes?	IF NO GO TO QU 39 No O (0)<br>Yes O (1)
37	If yes, are you currently receiving any of the following treatment/have you received any of the following advice by a doctor or health worker for your high blood sugar or diabetes?	Prescribed medication such as insulin O (1)<br>Advice/ treatment on special diet O (2)<br>Advice/treatment to lose weight O (3)<br>Advice/ treatment to stop smoking O (4)<br>Advice to start/do more exercise O (5)<br>None (6)
38	Are you currently taking any herbal or traditional remedies for your high blood sugar or diabetes	No O (0)<br>Yes O (1)
39	Has your blood pressure ever been measured?	No O (0)<br>Yes O (1)<br>Don’t know O (2)
40	Have you ever been told by a doctor or other health worker that you have high blood pressure or hypertension?	IF NO GO TO NEXT SECTION No O (0)<br>Yes O (1)
41	If yes, are you currently receiving any of the following treatment/have you received any of the following advice by a doctor or health worker to control your blood pressure	Prescribed medication O (1)<br>Advice to reduce salt intake O (2)<br>Advice/treatment to lose weight O (3)<br>Advice/ treatment to stop smoking O (4)<br>Advice to start/do more exercise O (5)<br>None (6)
42	Are you currently taking any herbal or traditional remedies for your high blood pressue?	No O (0)<br>Yes O (1)

REPRODUCTIVE HEALTH : WOMEN AGED 15 – 49 YEARS

1	Do you have any children?	IF NO GO TO Q4 No O (0)<br>Yes O (1)
2	Do you have any children under the age of 5?	No (0)<br>Yes (1)
3	How many children do you have today (excluding those who have died)?	☐☐
4	Did you have any pregnancies that ended before term (i.e. still birth, miscarriage or abortion)?	IF NO GO TO NEXT SECTION No O (0)<br>Yes O (1)
5	If yes, how many pregnancies ended before term?	☐☐

Guatemala National Disability Survey Case Control Questionnaire

**PREGNANCY CARE: WOMEN WITH CHILDREN AGED FIVE AND BELOW**		
	I would now like to ask you some questions about your youngest child born in the last 5 years. What is the name of the last child born in this period?	
1	Did you see anyone for antenatal care during this time?	IF NO GO TO Q3 No O (0)<br>Yes O (1)
2	Who did you see<br>(tick all that apply)	Non Trained Traditional Birth Attendant O (1)<br>Trained Traditional Birth Attendant O (2)<br>Auxilliary Nurse O (3)<br>Professional Nurse O (4)<br>Doctor O (5)<br>Traditional Healer O (6)<br>Nobody assisted O (7)<br>Other O (8)
2.1	Specify if other	✍
3	Where, specifically, did you give birth to [name]?	✍
4	Where did you give birth to [name]?	Home (your home) O (1)<br>Other home O (2)<br>Government Health Post O (3)<br>Government Health Centre O (4)<br>Government Hospital O (5)<br>IGSS supported hospital O (6)<br>Non Governmental Organization health center O (7)<br>Private Clinic O (8)<br>Private Hospital O (9)<br><br>Other (10)
4.1	Specify if other	✍
5	Did anyone assist in the delivery of [name]?	Non Trained Traditional Birth Attendant O (1)<br>Trained Traditional Birth Attendant O (2)<br>Auxilliary Nurse O (3)<br>Professional Nurse O (4)<br>Doctor O (5)<br>Traditional Healer O (6)<br>Nobody assisted O (7)<br>Other O (8)
5.1	Specify if other	✍
**VACCINE COVERAGE – PARTICIPANTS AGED 5-9 AND CAREGIVERS OF CHILDREN UNDER 10**		
1	(if participant is >15) Are you the primary caregiver of any children under 10?	No O (0)<br>Yes O (1)
2	(if participant is >15) What is the name of your youngest child born in this period?	✍
3	(if participant is >15) What is the child's age (years)	✍

Guatemala National Disability Survey Case Control Questionnaire		
4	Does the child have a vaccine card?	IF NO, GO TO NEXT SECTION No (0)<br>Yes (1)
5	If the child's vaccine card is available check all of the following that were received<br>(tick all that apply)	BCG (one dose) O (1)<br>Measles/Mumps/Rubella (one dose) O (2)<br>DPT (three doses) O (3)<br>Polio (three doses) O (4))
**REHABILITATION – PARTICIPANTS WITH DISABILITIES ONLY**		
A	Have you ever heard of [service]	IF NO GO TO Q2 No O (0)<br>Yes O (1)
B	Have you ever needed this type of service?	IF NO GO TO Q2 No O (0)<br>Yes O (1)
C	Have you ever received this type of service?	IF NO GO TO QE No O (0)<br>Yes O (1)
D	If yes, are you currently receiving this service?	IF NO GO TO QF No O (0)<br>IF YES GO TO Q2 Yes O (1)
E	If reported needing but not receiving the service, why is this?	Too expensive O (1)<br>Too far O (2)<br>Discrimination O (3)<br>Does not speak Spanish O (4)<br>Does not know sign language (5)<br>Does not know where to access O (6)<br>Service not available O (7)<br>Other O (8)
E.1	Specify if other	
F	If reported previously receiving this service but not receiving it now, why is this?	Too expensive O (1)<br>Too far O (2)<br>No longer available O (3)<br>Does not speak Spanish O (4)<br>Does not know sign language (5)<br>Does not know where to access O (6)<br>Not helping O (7)<br>Not satisfied O (8)<br>No longer needs it O (9)<br>Device broken and cannot repair O (10)<br>Other O (11)
F.1	Specify if other	
	Repeat for each of the following services:	

		A	B	C	D	E	F
1	Medical rehabilitation						
2	Assistive Device Services						
3	Specialist education services						
4	Vocational Training						
5	Counselling						
6	Welfare Services						

Guatemala National Disability Survey Case Control Questionnaire							
		A	B	C	D	E	F
7	Health Information						
8	Traditional/faith healing						
9	Legal Advice						
10	Specialist health services						
11	Community Based Rehabilitation						

ASSISTIVE DEVICES – PARTICIPANTS WITH DISABILITIES ONLY		
	I am going to read you a list of assistive devices. For each please tell me if you use it, need it but don't use it, don't need it, or don’t know what it is.	
1	Glasses	Use it O (1)<br>Need it but don’t use it O (2)<br>Don’t need it O (3)<br>Don’t know what it is O (4)
2	Magnifying Glass	Use it O (1)<br>Need it but don’t use it O (2)<br>Don’t need it O (3)<br>Don’t know what it is O (4)
3	Telescopic lenses	Use it O (1)<br>Need it but don’t use it O (2)<br>Don’t need it O (3)<br>Don’t know what it is O (4)
4	Enlarged print	Use it O (1)<br>Need it but don’t use it O (2)<br>Don’t need it O (3)<br>Don’t know what it is O (4)
5	Braille	Use it O (1)<br>Need it but don’t use it O (2)<br>Don’t need it O (3)<br>Don’t know what it is O (4)
6	Hearing aid	Use it O (1)<br>Need it but don’t use it O (2)<br>Don’t need it O (3)<br>Don’t know what it is O (4)
7	Wheelchair	Use it O (1)<br>Need it but don’t use it O (2)<br>Don’t need it O (3)<br>Don’t know what it is O (4)
8	Crutches	Use it O (1)<br>Need it but don’t use it O (2)<br>Don’t need it O (3)<br>Don’t know what it is O (4)
9	White cane	Use it O (1)<br>Need it but don’t use it O (2)<br>Don’t need it O (3)<br>Don’t know what it is O (4)

Guatemala National Disability Survey Case Control Questionnaire		
10	Walking stick	Use it O (1)<br>Need it but don't use it O (2)<br>Don't need it O (3)<br>Don't know what it is O (4)
11	Guide (person or dog)	Use it O (1)<br>Need it but don't use it O (2)<br>Don't need it O (3)<br>Don't know what it is O (4)
12	Standing Frame	Use it O (1)<br>Need it but don't use it O (2)<br>Don't need it O (3)<br>Don't know what it is O (4)
13	Prosthesis	Use it O (1)<br>Need it but don't use it O (2)<br>Don't need it O (3)<br>Don't know what it is O (4)
14	Orthosis	Use it O (1)<br>Need it but don't use it O (2)<br>Don't need it O (3)<br>Don't know what it is O (4)
15	Other – specify	✍
15.1	Other	Use it O (1)<br>Need it but don't use it O (2)<br>Don't need it O (3)<br>Don't know what it is O (4)
**PARTICIPATION RESTRICTIONS – ALL PARTICIPANTS**		
	Do you have any difficulty performing the following activities in your current environment? This means even with the help of assistive devices or another person	
1	Washing oneself	No difficulty O (1)<br>Moderate difficulty O (2)<br>Severe difficulty O (3)<br>Unable to do O (4)<br>Don't know O (5)
2	Care of body parts, teeth, nails and hair	No difficulty O (1)<br>Moderate difficulty O (2)<br>Severe difficulty O (3)<br>Unable to do O (4)<br>Don't know O (5)
3	Toileting	No difficulty O (1)<br>Moderate difficulty O (2)<br>Severe difficulty O (3)<br>Unable to do O (4)<br>Don't know O (5)
4	Dressing and undressing	No difficulty O (1)<br>Moderate difficulty O (2)<br>Severe difficulty O (3)<br>Unable to do O (4)<br>Don't know O (5)

Guatemala National Disability Survey Case Control Questionnaire		
5	Eating and drinking	No difficulty O (1)<br>Moderate difficulty O (2)<br>Severe difficulty O (3)<br>Unable to do O (4)<br>Don't know O (5)
6	Shopping (9+)	No difficulty O (1)<br>Moderate difficulty O (2)<br>Severe difficulty O (3)<br>Unable to do O (4)<br>Don't know O (5)
7	Preparing meals (9+)	No difficulty O (1)<br>Moderate difficulty O (2)<br>Severe difficulty O (3)<br>Unable to do O (4)<br>Don't know O (5)
8	Doing housework (9+)	No difficulty O (1)<br>Moderate difficulty O (2)<br>Severe difficulty O (3)<br>Unable to do O (4)<br>Don't know O (5)
9	Taking care of personal objects (9+)	No difficulty O (1)<br>Moderate difficulty O (2)<br>Severe difficulty O (3)<br>Unable to do O (4)<br>Don't know O (5)
10	Taking care of others	No difficulty O (1)<br>Moderate difficulty O (2)<br>Severe difficulty O (3)<br>Unable to do O (4)<br>Don't know O (5)
11	Making friends and maintaining friendships	No difficulty O (1)<br>Moderate difficulty O (2)<br>Severe difficulty O (3)<br>Unable to do O (4)<br>Don't know O (5)
12	Interacting with persons of authority (16+)	No difficulty O (1)<br>Moderate difficulty O (2)<br>Severe difficulty O (3)<br>Unable to do O (4)<br>Don't know O (5)
13	Interacting with strangers	No difficulty O (1)<br>Moderate difficulty O (2)<br>Severe difficulty O (3)<br>Unable to do O (4)<br>Don't know O (5)
14	Creating and maintaining family relationships	No difficulty O (1)<br>Moderate difficulty O (2)<br>Severe difficulty O (3)<br>Unable to do O (4)<br>Don't know O (5)

Guatemala National Disability Survey Case Control Questionnaire		
15	Creating and maintaining intimate relationships (16+)	No difficulty O (1)<br>Moderate difficulty O (2)<br>Severe difficulty O (3)<br>Unable to do O (4)<br>Don't know O (5)
16	Going to school and studying (<17)	No difficulty O (1)<br>Moderate difficulty O (2)<br>Severe difficulty O (3)<br>Unable to do O (4)<br>Don't know O (5)
17	Getting and keeping a job (16+)	No difficulty O (1)<br>Moderate difficulty O (2)<br>Severe difficulty O (3)<br>Unable to do O (4)<br>Don't know O (5)
18	Handling income and payments (16+)	No difficulty O (1)<br>Moderate difficulty O (2)<br>Severe difficulty O (3)<br>Unable to do O (4)<br>Don't know O (5)
19	Joining clubs and organisations (16+)	No difficulty O (1)<br>Moderate difficulty O (2)<br>Severe difficulty O (3)<br>Unable to do O (4)<br>Don't know O (5)
20	Participating in recreation and leisure activities (16+)	No difficulty O (1)<br>Moderate difficulty O (2)<br>Severe difficulty O (3)<br>Unable to do O (4)<br>Don't know O (5)
21	Participating in religious/spiritual activities (16+)	No difficulty O (1)<br>Moderate difficulty O (2)<br>Severe difficulty O (3)<br>Unable to do O (4)<br>Don't know O (5)
22	Participating in political life and citizenship (16+)	No difficulty O (1)<br>Moderate difficulty O (2)<br>Severe difficulty O (3)<br>Unable to do O (4)<br>Don't know O (5)
**ENVIRONMENT**		
	In the last twelve months, how often:	
1	Has the availability/accessibility of transportation been a problem for you?	Daily O (1)<br>Weekly O (2)<br>Monthly O (3)<br>Less than monthly O (4)<br>GO TO NEXT QUESTION Never O (5)
1.1	When this problem occurs is it a big problem or a little problem?	Big problem O (1)<br>Little problem O (2)

Guatemala National Disability Survey Case Control Questionnaire		
2	Has the natural environment – temperature, terrain, climate – made it difficult to do what you want or need to do?	Daily O (1)<br>Weekly O (2)<br>Monthly O (3)<br>Less than monthly O (4)<br>GO TO NEXT QUESTION Never O (5)
2.1	When this problem occurs is it a big problem or a little problem?	Big problem O (1)<br>Little problem O (2)
3	Have other things in your surroundings – lighting, noise, crowds, etc – made it difficult to do what you want or need to do?	Daily O (1)<br>Weekly O (2)<br>Monthly O (3)<br>Less than monthly O (4)<br>GO TO NEXT QUESTION Never O (5)
3.1	When this problem occurs is it a big problem or a little problem?	Big problem O (1)<br>Little problem O (2)
4	Has the information you wanted or needed not been available in a format you can use or understand?	Daily O (1)<br>Weekly O (2)<br>Monthly O (3)<br>Less than monthly O (4)<br>GO TO NEXT QUESTION Never O (5)
4.1	When this problem occurs is it a big problem or a little problem?	Big problem O (1)<br>Little problem O (2)
5	Has the availability of health care services and medical care been a problem for you?	Daily O (1)<br>Weekly O (2)<br>Monthly O (3)<br>Less than monthly O (4)<br>GO TO NEXT QUESTION Never O (5)
5.1	When this problem occurs is it a big problem or a little problem?	Big problem O (1)<br>Little problem O (2)
6	Did you need someone else’s help in your home and could not get it easily?	Daily O (1)<br>Weekly O (2)<br>Monthly O (3)<br>Less than monthly O (4)<br>GO TO NEXT QUESTION Never O (5)
6.1	When this problem occurs is it a big problem or a little problem?	Big problem O (1)<br>Little problem O (2)
7	Did you need someone else’s help at school or work and could not get it easily?	Daily O (1)<br>Weekly O (2)<br>Monthly O (3)<br>Less than monthly O (4)<br>GO TO NEXT QUESTION Never O (5)
7.1	When this problem occurs is it a big problem or a little problem?	Big problem O (1)<br>Little problem O (2)
8	Have other people’s attitudes toward you been a problem at home?	Daily O (1)<br>Weekly O (2)<br>Monthly O (3)<br>Less than monthly O (4)<br>GO TO NEXT QUESTION Never O (5)
8.1	When this problem occurs is it a big problem or a little problem?	Big problem O (1)<br>Little problem O (2)

Guatemala National Disability Survey Case Control Questionnaire		
9	Have other people's attitudes toward you been a problem at school or work?	Daily O (1)<br>Weekly O (2)<br>Monthly O (3)<br>Less than monthly O (4)<br>GO TO NEXT QUESTION Never O (5)
9.1	When this problem occurs is it a big problem or a little problem?	Big problem O (1)<br>Little problem O (2)
10	Did you experience prejudice or discrimination?	Daily O (1)<br>Weekly O (2)<br>Monthly O (3)<br>Less than monthly O (4)<br>GO TO NEXT QUESTION Never O (5)
10.1	When this problem occurs is it a big problem or a little problem?	Big problem O (1)<br>Little problem O (2)
11	Did the policies and rules of businesses and organizations make problems for you?	Daily O (1)<br>Weekly O (2)<br>Monthly O (3)<br>Less than monthly O (4)<br>GO TO NEXT QUESTION Never O (5)
11.1	When this problem occurs is it a big problem or a little problem?	Big problem O (1)<br>Little problem O (2)
12	Did government programs and policies make it difficult to do what you want or need to do?	Daily O (1)<br>Weekly O (2)<br>Monthly O (3)<br>Less than monthly O (4)<br>GO TO NEXT QUESTION Never O (5)
12.1	When this problem occurs is it a big problem or a little problem?	Big problem O (1)<br>Little problem O (2)
